# Influence of Genistein on Hepatic Lipid Metabolism in an In Vitro Model of Hepatic Steatosis

**DOI:** 10.3390/molecules26041156

**Published:** 2021-02-22

**Authors:** Lena Seidemann, Anne Krüger, Victoria Kegel-Hübner, Daniel Seehofer, Georg Damm

**Affiliations:** 1 Department of Hepatobiliary Surgery and Visceral Transplantation, University Hospital, Leipzig University, 04103 Leipzig, Germany; lena.seidemann@medizin.uni-leipzig.de (L.S.); victoria.kegel@yahoo.de (V.K.-H.); daniel.seehofer@medizin.uni-leipzig.de (D.S.); 2 Saxonian Incubator for Clinical Translation (SIKT), Leipzig University, 04103 Leipzig, Germany; 3 Department of General, Visceral and Transplantation Surgery, Charité University Medicine Berlin, 13353 Berlin, Germany; anne.krueger@med.uni-greifswald.de

**Keywords:** liver, primary human hepatocytes, steatosis, NAFLD, NASH, Genistein, PPARα, SREBP-1c

## Abstract

Nonalcoholic fatty liver disease (NAFLD) is among the leading causes of end-stage liver disease. The impaired hepatic lipid metabolism in NAFLD is exhibited by dysregulated PPARα and SREBP-1c signaling pathways, which are central transcription factors associated with lipid degradation and de novo lipogenesis. Despite the growing prevalence of this disease, current pharmacological treatment options are unsatisfactory. Genistein, a soy isoflavone, has beneficial effects on lipid metabolism and may be a candidate for NAFLD treatment. In an in vitro model of hepatic steatosis, primary human hepatocytes (PHHs) were incubated with free fatty acids (FFAs) and different doses of genistein. Lipid accumulation and the cytotoxic effects of FFAs and genistein treatment were evaluated by colorimetric and enzymatic assays. Changes in lipid homeostasis were examined by RT-qPCR and Western blot analyses. PPARα protein expression was induced in steatotic PHHs, accompanied by an increase in CPT1L and ACSL1 mRNA. Genistein treatment increased PPARα protein expression only in control PHHs, while CPTL1 and ACSL1 were unchanged and PPARα mRNA was reduced. In steatotic PHHs, genistein reversed the increase in activated SREBP-1c protein. The model realistically reflected the molecular changes in hepatic steatosis. Genistein suppressed the activation of SREBP-1c in steatotic hepatocytes, but the genistein-mediated effects on PPARα were abolished by high hepatic lipid levels.

## 1. Introduction

Nonalcoholic fatty liver disease (NAFLD) is the most common cause of chronic liver disease worldwide, with a global prevalence of approximately 25% [1,2]. It is considered the hepatic manifestation of metabolic syndrome and comprises a wide spectrum of liver impairments, starting with hepatic steatosis, which can progress to more severe liver damage, e.g., steatohepatitis (NASH), fibrosis and cirrhosis [3]. Predictably, NAFLD is becoming one of the leading indications for liver transplantation in the United States and in Europe [4,5]. Moreover, the growing incidence of hepatic steatosis in donor organs aggravates the problem of organ shortage [6].

Hepatic steatosis, the first stage of NAFLD, is marked by intracellular accumulation of triglycerides due to increased uptake of free fatty acids (FFAs), augmented de novo lipogenesis and impaired fatty acid β-oxidation [7,8,9]. The underlying molecular mechanisms are not fully understood but are currently being extensively studied, with the goal of a possible drug target to enable pharmacological NAFLD treatment [10].

One key player in hepatic lipid metabolism is peroxisome proliferator-activated receptor alpha (PPARα), a ligand-activated transcription factor that is highly expressed in the liver [11]. The binding of FFAs to PPARα induces the transcription of several genes involved in mitochondrial FFA β-oxidation and FFA transport [12]. Intracellular processing of long-chain fatty acids has to be preceded by their activation to acyl-coenzyme A (acyl-CoA) via long-chain acyl-CoA synthetases (ACSL). ACSL1 is the predominant isoform in the liver and a target gene of PPARα [13,14]. After esterification by ACSL1, long-chain acyl-CoA can enter β-oxidation via active transport into the mitochondria. The rate-limiting step of this process is transport through the outer mitochondrial membrane by carnitine palmitoyltransferase 1L (CPT1L), another transcription product of PPARα [14,15].

Sterol regulatory element-binding protein 1c (SREBP-1c) is a central transcriptional regulator of hepatic de novo lipogenesis [9]. Insulin induces the expression of an inactive SREBP-1c precursor bound to the endoplasmic reticulum (ER) and likewise promotes its activation by proteolytic cleavage [16]. SREBP-1c then induces the expression of enzymes synthesizing fatty acids, i.e., acetyl-CoA carboxylase (ACC) and fatty acid synthase (FASN), which together catalyze the synthesis of palmitic acid [9,17].

Liver biopsies of NAFLD patients have shown increased expression of SREBP-1c and its target genes and decreased expression of PPARα and CPT1L [18,19]. To compensate for the reduced mitochondrial β-oxidation, the upregulated gene expression of enzymes engaged in peroxisomal β-oxidation and microsomal ω-oxidation was observed [19]. These pathways are less effective in breaking down fatty acids and lead to an accumulation of reactive oxygen species (ROS) as byproducts [20]. Oxidative stress likely plays a crucial role in inflammation and hepatocyte damage observed in NAFLD [21]. This proinflammatory situation may be even more enhanced by the inhibition of PPARα, a known negative regulator of inflammation and acute phase response, thus paving the way for disease progression from hepatic steatosis to steatohepatitis [22,23].

To date, no drug for NAFLD therapy is available. NAFLD management consists of a multimodal approach, similar to the treatment for metabolic syndrome, mainly focusing on lifestyle changes, i.e., improving diet and physical activity. Pharmacological treatments aim for the amelioration of associated metabolic disorders, such as hypertriglyceridemia [24].

Soy isoflavones, so-called phytoestrogens, have recently gained interest because of their beneficial effects on menopausal complaints and have also been proposed to positively affect metabolic syndrome and associated diseases due to their antioxidative and hypolipidemic properties [25,26,27]. An 8-week oral supplementation with genistein, the most abundant isoflavone of soybean, reduced oxidative and inflammatory indices alongside improvements in fat metabolism in NAFLD patients [28]. Genistein treatment further attenuated the development of steatosis in rodent in vitro and in vivo models [29,30,31]. This improvement was accompanied by a reversal of the FFA-induced downregulation of PPARα and its downstream targets, such as CPT1L [29,31].

Primary human hepatocytes (PHHs) are still considered the gold standard for the creation of in vitro liver models [10]. A recent publication has underlined the protective effects of genistein against NAFLD also in PHHs [32]. However, in the above-mentioned studies, genistein was administered prior to or throughout a steatotic treatment, thus underlining its role in NAFLD prevention [29,30,31,32]. In our study, we sought to investigate the effects of genistein on hepatic lipid metabolism after manifestation of steatosis. Therefore, PHHs were treated with FFAs to induce in vitro steatosis and treated with different concentrations of genistein. The in vitro steatosis and the effect of genistein on steatotic and control PHHs were investigated by evaluating lipid storage, cytotoxic effects and changes in lipid homeostasis. Our results show that our in vitro steatosis model was characterized by high lipid accumulation accompanied by mild lipotoxic effects and a lipid-related shift in signaling towards lipid metabolism. Treatment of steatotic and control PHHs with genistein revealed that a high lipid load eliminated the beneficial effects of genistein on lipid metabolism, which were clearly visible in control PHHs.

## 2. Results

### 2.1. Incubation of PHHs with Free Fatty Acids Leads to Intracellular Lipid Accumulation

**Figure 1:**
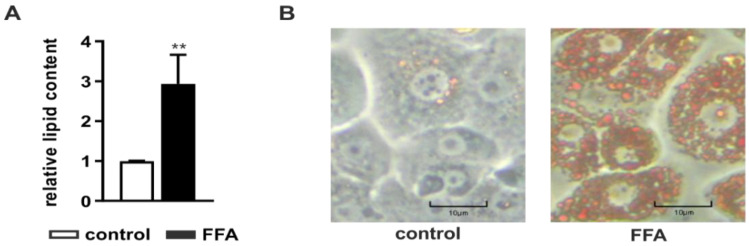
Amount of neutral lipids in primary human hepatocytes (PHHs) after treatment with free fatty acids (FFAs). PHHs were treated with 1 mM FFAs for 24 h and lipid levels were quantified using the Oil Red O assay normalized to the protein amount measured by sulforhodamine B (SRB) assay. (**A**) Evaluation of steatosis in FFA-treated PHHs in comparison to control. Data are shown as the mean + SD, *n* = 5, paired t-test, *p* < 0.01 (**). (**B**) Microscopic evaluation of the lipid accumulation in representative PHH cultures (magnification 200×), the scale bar is 10 µm.

After PHH isolation and initial adherence overnight, steatosis was induced by incubation with 1 mM FFAs consisting of a mixture of oleate and palmitate in a 2:1 ratio for 24 h. The intracellular lipid accumulation was determined with the Oil Red O assay normalized to the protein content measured with the sulforhodamine B (SRB) assay. FFA-treated PHHs showed a significant accumulation of neutral lipids (Figure 1A) visible in a larger number and size of intracellular lipid droplets (Figure 1B).

### 2.2. The Hepatocellular Steatosis Model Shows Only Mild Lipotoxic Effects

**Figure 2:**
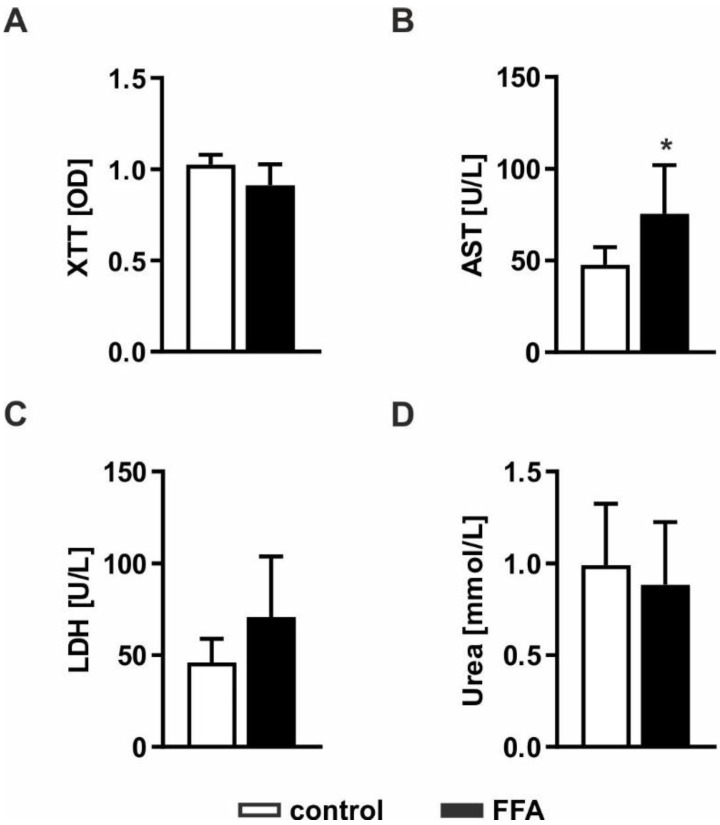
Evaluation of lipotoxicity on FFA-treated PHHs. PHHs were treated with 1 mM FFAs for 24 h and steatotic and control PHHs were investigated for (**A**) cell activity measured by the conversion of XTT (2,3-bis-(2-methoxy-4-nitro-5-sulfophenyl)-2*H*-tetrazolium-5-carboxanilid); (**B**) enzyme activities of AST (aspartate transaminase) and (**C**) LDH (lactate dehydrogenase) determined in cell culture supernatants to evaluate disruptions of cell membrane integrity and (**D**) metabolic capacity examined by quantification of their urea production. Data are shown as the mean + SD, *n* = 5, paired t-test, *p* < 0.05 (*).

Severe lipid accumulation can lead to lipotoxic effects and cell death. Therefore, we investigated whether FFA treatment leads to impaired cellular functions and cell loss. Pathological effects were evaluated by measurement of cell activity using XTT (2,3-bis-(2-methoxy-4-nitro-5-sulfophenyl)-2*H*-tetrazolium-5-carboxanilid) assays, the ability to produce urea and impaired membrane integrity by measurement of LDH (lactate dehydrogenase) and AST (aspartate transaminase) release (Figure 2). Determination of cell activity showed no difference between FFA-treated PHHs and control cells, indicating comparable metabolic activity in both cultures (Figure 2A). Extracellular AST activity was significantly increased, whereas LDH activity showed an increase only by trend (Figure 2B,C). Determination of urea production showed no influence on metabolic capacities in the steatotic hepatocytes (Figure 2D). Taken together, the lipid accumulation in steatotic PHHs led to mild lipotoxic effects.

### 2.3. Transcription of PPARα Downstream Signaling Targets Is Increased in Steatosis

**Figure 3:**
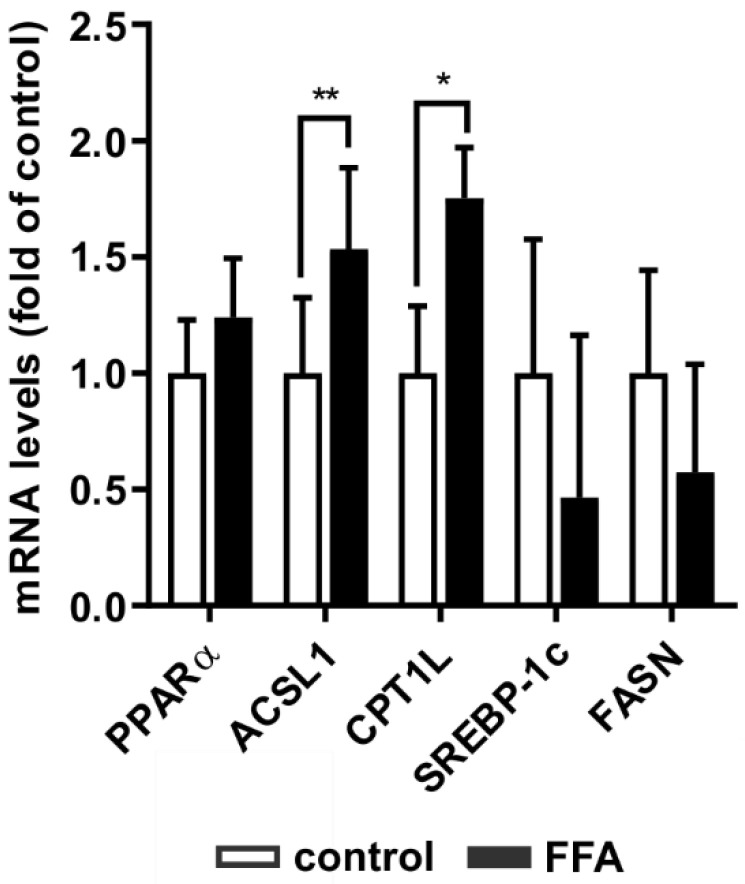
Adaption of gene expression of key players of lipid homeostasis in steatotic PHHs. PHHs were treated with 1 mM FFAs for 24 h and relative mRNA expression levels of gene targets were determined using RT-qPCR. Investigation of genes centrally involved in lipid catabolism (PPARα, ACSL1 and CPT1L) and anabolism (SREBP-1c and FASN) in PHHs compared to control. Data are shown as the mean + SD, *n* = 5, paired t-test, statistical analyses were conducted on ΔC_T_ values, *p* ≤ 0.05 (*), *p* < 0.01 (**).

**Figure 4:**
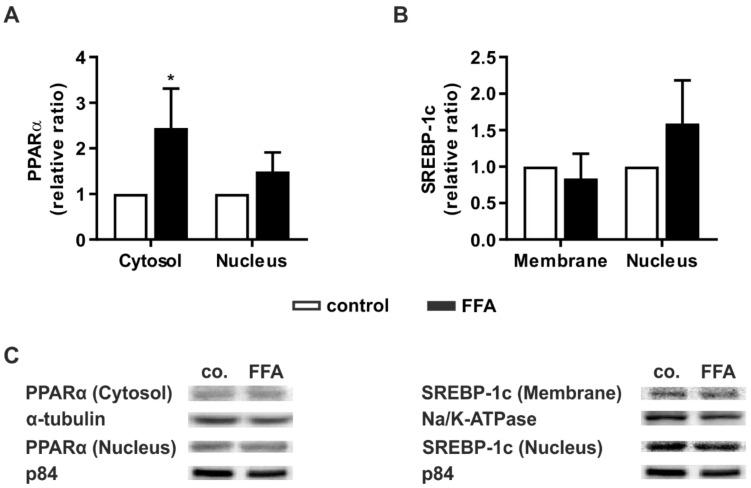
Activation of key signaling pathways in lipid homeostasis in steatotic hepatocytes. PHHs were treated with 1 mM FFAs for 24 h and protein levels of PPARα and SREBP-1c were investigated. Western blot analyses were performed after protein extraction from different subcellular fractions of FFA-treated PHHs and control. Densitometric measurements of (**A**) cytosolic PPARα normalized to the expression levels of α-tubulin, nucleic PPARα normalized to p84, (**B**) ER membrane-bound SREBP-1c normalized to Na^+^/K^+^-ATPase and nucleic SREBP-1c normalized to p84 expression. Data are shown as the mean + SD, *n* = 5, paired t-test, *p* ≤ 0.05 (*). (**C**) Representative Western blot images show the specific bands of PPARα, α-tubulin, p84, SREBP-1c, and Na^+^/K^+^-ATPase in control and steatotic PHHs.

NAFLD is characterized by changes in lipid homeostasis resulting in dysregulated lipid degradation, impaired FFA β-oxidation and augmented de novo lipogenesis [7,8,9]. In hepatocytes, PPARα is a transcription factor responsible for the expression of target genes of lipid metabolism [11,12]. In contrast, SREBP-1c is a transcription factor that is responsible for the expression of lipogenesis genes [9]. Therefore, we analyzed the influence of in vitro lipid accumulation on key players in lipid homeostasis at the transcript level using RT-qPCR (Figure 3) and at the protein level using Western blot analyses (Figure 4).

The transcription factor PPARα is a major mediator of fatty acid degradation. It induces the transcription of ACSL1 and CPT1L, two enzymes that catalyze necessary steps prior to fatty acid β-oxidation. FFA-treated hepatocytes showed increased mRNA levels of ACSL1 and CPT1L, while PPARα mRNA showed only a slight tendency to increase (Figure 3). SREBP-1c and its transcription target FASN, both involved in fatty acid synthesis, did not show any statistically significant transcriptional changes. Thus, steatotic hepatocytes showed an adaption of lipid homeostasis towards catabolism, while promoters of de novo lipogenesis did not adjust to the increased lipid load.

### 2.4. Steatotic Treatment Increases the Level of Cytosolic PPARα

For validation of the transcript data at the protein level, we performed Western blot analyses. Additionally, we investigated whether increased levels of key signaling proteins correlate with a higher activation of these mediators. Therefore, the protein levels of the activated forms of PPARα and SREBP-1c were measured.

PPARα shuttles between the cytosol and nucleus, and its concentration in the latter is crucial for its action as a transcription factor [33]. After FFA treatment, the protein levels of PPARα in both cellular compartments were determined relative to untreated PHHs. In both compartments, PPARα showed higher levels in steatotic hepatocytes, but this increase was only significant for cytosolic PPARα (Figure 4A).

The precursor form of SREBP-1c is bound to the endoplasmic reticulum (ER) membrane. Activation occurs through proteolytic cleavage. Cleaved SREBP-1c then translocates to the nucleus [34]. The protein levels of ER membrane-bound or nucleic SREBP-1c did not differ between the vehicle-treated and FFA-treated PHH groups (Figure 4B). Taken together, the protein data confirmed the transcript data. Additionally, we observed hints of increased activation of the metabolic pathway by activation of PPARα and expression of its downstream targets.

### 2.5. Genistein Administration Causes Hepatotoxicity Only in High Doses

**Figure 5:**
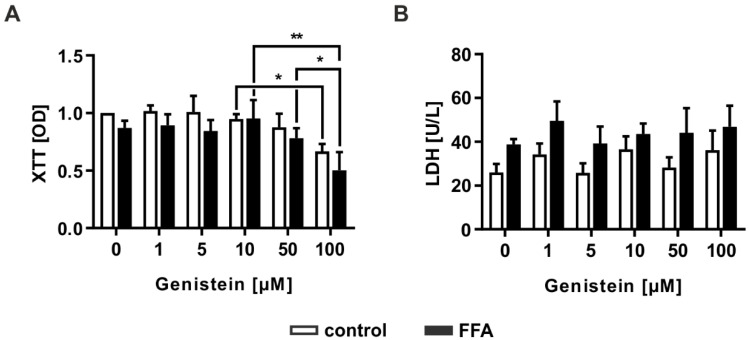
Evaluation of the cytotoxic effect of different concentrations of the soy isoflavone genistein on FFA-treated and control PHHs. PHHs were treated with 1 mM FFAs for 24 h, followed by 24 h of treatment with genistein (0, 1, 5, 10, 50, and 100 µM). Toxicity was measured by evaluation of (**A**) cell activity determined with the XTT (2,3-bis-(2-methoxy-4-nitro-5-sulfophenyl)-2*H*-tetrazolium-5-carboxanilid) assay and (**B**) LDH (lactate dehydrogenase) release as a marker for impaired cell integrity. Data are shown as the mean + SD, *n* = 3, two-way ANOVA and post hoc Tukey or Sidak test, *p* ≤ 0.05 (*), *p* < 0.01 (**). Selected comparisons are shown; for details on the statistical evaluation, see Table S1A, Supplementary Materials.

Genistein is a natural compound in soybean and is described as a PPARα agonist [35]. Therefore, it can decrease hepatic lipid content by increasing lipid metabolism [29]. For evaluation of the toxic effects of genistein, it was administered to FFA- or vehicle-treated PHHs at different concentrations (0, 1, 5, 10, 50, and 100 µM). The cytotoxic effects of the substance were investigated by measuring cellular metabolic activity reflecting cell viability using the XTT assay and by analyzing LDH activity reflecting a decrease in membrane integrity due to cytotoxic effects. Genistein treatment demonstrated impaired cell viability in PHHs independent of FFA treatment at the highest administered genistein concentration of 100 µM (Figure 5A). Nonetheless, the addition of genistein did not augment the release of LDH into the cell culture supernatant, as happens in necrosis or late apoptosis (Figure 5B). However, the induction of apoptosis at high concentrations could not be excluded, indicating that further results for 100 µM genistein should be discussed critically.

### 2.6. Genistein Induces PPARα Activation Only in Control PHHs

**Figure 6:**
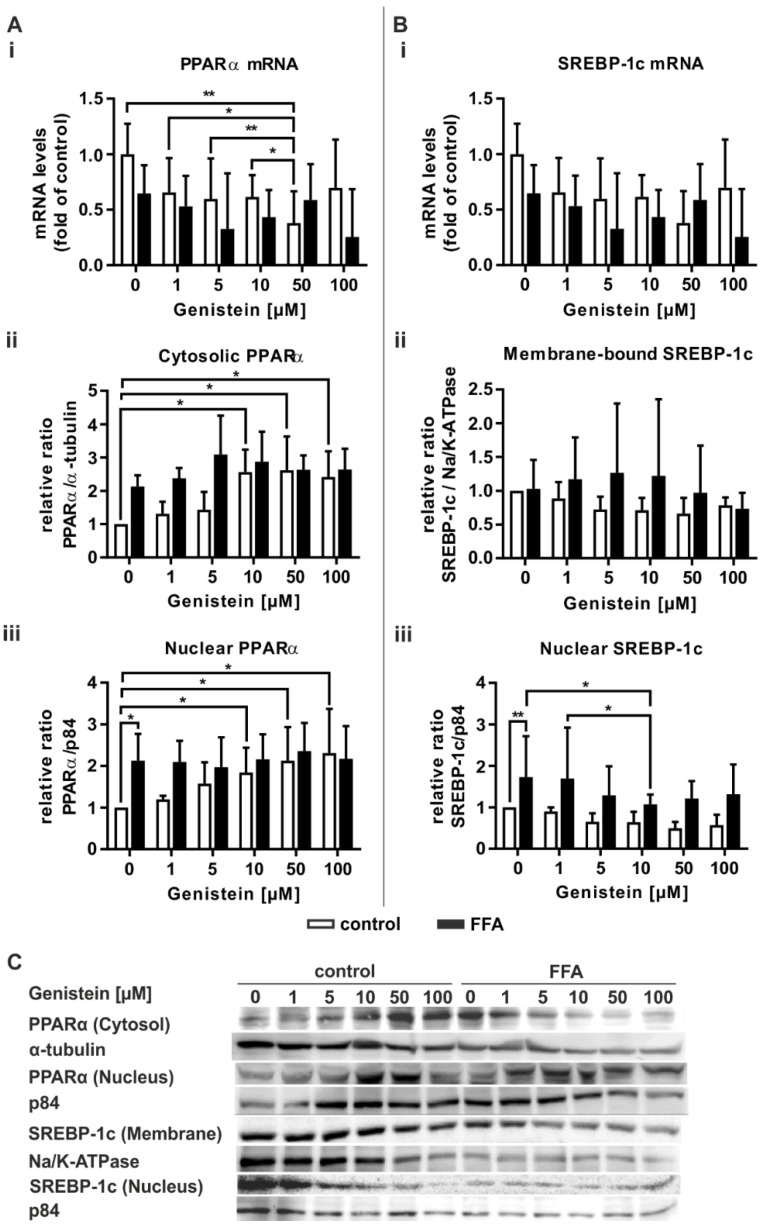
Transcriptional and translational response of genistein-treated steatotic PHHs on (**A**) PPARα and (**B**) SREBP-1c, two central regulators of hepatic lipid homeostasis. PHHs were treated with 1 mM FFAs for 24 h, followed by 24 h of additive treatment with genistein (0, 1, 5, 10, 50, and 100 µM). (**Ai**) PPARα mRNA levels were determined by RT-qPCR; (**Aii**) cytosolic and; (**Aiii**) nucleic PPARα protein levels were assessed by Western blot (α-tubulin and p84 served as the respective reference proteins). (**Bi**) Relative mRNA expression levels of SREBP-1c as measured by RT-qPCR; (**Bii**) densitometric measurements of ER membrane-bound SREBP-1c normalized to Na^+^/K^+^-ATPase and (**Biii**) nucleic SREBP-1c normalized to p84 expression. Data are shown as the mean + SD, *n* = 5 for RT-qPCR data, *n* = 4 for Western blot measurements, two-way ANOVA with Tukey or Sidak post hoc test. *p* ≤ 0.05 (*), *p* < 0.01 (**). Selected comparisons are shown; for details on the statistical evaluation, see Table S1B, Supplementary Materials. (**C**) Representative Western blot images show the specific bands of PPARα, α-tubulin, p84, SREBP-1c, and Na^+^/K^+^-ATPase in control and steatotic PHHs after genistein treatment.

Genistein acts as a PPARα agonist [35]. Therefore, we tested the ability of genistein to influence lipid-associated signaling pathways. Treatment of control PHHs with genistein led to a concentration-dependent decrease in PPARα mRNA expression, while no change in PPARα expression in steatotic PHHs was observable (Figure 6Ai). Both PHH groups featured no further change in the mRNA levels of the transcriptional targets of PPARα (Figure S1, Supplementary Materials). Only the highest genistein concentration of 100 µM, which had shown cytotoxic effects in the XTT assay (Figure 5A), led to a decrease in ACSL1 mRNA (Figure S1B). A concentration-dependent increase in PPARα activation was observable in an increase in cytosolic and nuclear PPARα protein in control PHHs (Figure 6Aii,Aiii). In contrast, PPARα activation in steatotic PHHs was initially increased and showed no further rise that was dependent on the genistein dose.

In regard to SREBP-1c mRNA and its downstream target FASN, no changes in its expression were observable due to genistein treatment (Figure 6Bi and Figure S1C). Notably, the concentration-dependent rise in the variation in the protein level of membrane-bound SREBP-1c suggests a highly donor-dependent effect on SREBP-1c expression (Figure 6Bii). However, SREBP-1c activity was decreased in genistein-treated steatotic hepatocytes, with a significant result for 10 µM genistein (Figure 6Biii).

In summary, the PPARα agonistic and thus steatosis-counteracting properties of genistein were only observed in control PHHs at the protein level. In contrast, a downregulation of fatty acid synthesis mediating SREBP-1c was only seen in steatotic PHHs, although only after treatment with 10 µM genistein

## 3. Discussion

To date, the growing global disease burden of NAFLD has not been remedied by an adequate pharmaceutical treatment [36]. Complex mechanisms, including dyslipidemia, hyperinsulinemia and inflammation, drive the development of hepatic steatosis and fuel its progression up to the point of cirrhosis and end-stage liver disease [37]. Drugs currently used in NAFLD management only treat individual etiological factors that contribute to its onset and progression [24]. In addition, some of them, such as PPARα agonistic fibrates, have been reported to exert hepatotoxicity themselves, which is a major drawback in NAFLD treatment [38]. The phytoestrogen genistein is a natural compound contained in soybean and has been described as a PPARα agonist [35]. Epidemiological data conclude that a higher soy food intake is associated with a lower prevalence of NAFLD [39].

Therefore, this study aimed to investigate the potential of genistein as a therapeutic treatment to improve steatosis in humans.

### 3.1. Induction of Steatosis Is Accompanied by Mild Lipotoxic Effects

The applied in vitro model, using oleate and palmitate in a 2:1 ratio, aims at the imitation of benign chronic steatosis [40]. As intended, treatment of PHHs with FFAs led to considerable intracellular lipid accumulation, which is in line with findings of our group and others [40,41]. This increased intracellular lipid content did not lead to substantial lipotoxic effects. Only one of the four different parameters that we evaluated as indicators of cell viability showed a significant response. The extracellular enzymatic activity of AST was slightly increased in steatotic hepatocytes. Transaminase measurements are a common way to assess liver injury in clinical and experimental settings. As this readout shows primarily a disturbance in membrane integrity very sensitively, a slight elevation in the context of cell viability has to be interpreted carefully. Instead, the determination of further hepatic viability parameters, such as metabolic competency via urea measurement and of cell activity via the tetrazolium salt assay, has been shown to yield a more comprehensive result for cell viability [42]. Induction of steatosis did not lead to alterations in those two parameters, as was already reported by our group [41].

### 3.2. FFA-Treated PHHs Exhibit Changes in Lipid Catabolism and Anabolism Pathways Analogous to the First Stages of NAFLD

The nuclear transcription factor PPARα is a key regulator of hepatic lipid degradation. It is activated by diverse exogenous and endogenous ligands, i.e., dietary fatty acids such as oleate and palmitate [12,43]. Other cell culture experiments conducted with PHHs or hepatic cell lines revealed an upregulation of PPARα or its transcription target CPT1L after 24 h of FFA treatment [44,45]. Our study confirms that FFA treatment of PHHs resulted in PPARα activation, as seen by an increase in the cytosolic protein and the PPARα transcription targets ACSL1 and CPT1L. This effect was even more enhanced 24 h later, when the steatotic hepatocytes without genistein treatment also showed a significant increase in the nucleic PPARα protein (Figure 6Aiii). ACSL1 and CPT1L encode proteins involved in the preliminary steps of FFA breakdown by mitochondrial β-oxidation [15]. These processes reflect the hepatocytes’ intention to reduce the escalated intracellular lipid load.

A further physiological requirement to limit lipid storage is the downregulation of de novo lipogenesis. Unsaturated fatty acids regulate SREBP-1c, a major transcription factor in lipid synthesis, not only at the transcriptional level but also by accelerating its mRNA turnover and restricting the proteolytic processing of the protein. Proteolytic cleavage of the ER membrane-bound precursor is suppressed, thus reducing the release of the active nuclear SREBP-1c isoform and diminishing its transcriptional activity [46]. After 24 h of FFA treatment, we observed a slight but insignificant decrease in the mRNA levels of SREBP-1c and FASN in the steatotic PHHs compared to the control. In contrast to the decrease in SREBP-1c mRNA, we observed an insignificant increase in the activated SREBP-1c protein, which became significant 24 h later in steatotic hepatocytes that were not supplemented with genistein (Figure 6Biii). An insignificant increase in FASN transcription is in line with SREBP-1c activation but vanishes with donor variances. Investigations on HepG2 cells incubated under comparable conditions of FFA treatment, although for 14 h, resulted in similar SREBP-1c and FASN expressions to those observed by us, thus indicating a gradual change in their trend of expression from short-term to long-term incubation times as also suggested by us [47]. Studies on human liver biopsies have reported increased SREBP-1c expression in samples from NAFLD patients [18]. Reduced PPARα expression is documented in liver biopsies featuring NASH or fibrosis, while those of simple steatotic livers do not differ from healthy liver tissue [48]. In our model of benign steatosis, we did not detect changes in PPARα mRNA levels, although a slight decrease in PPARα mRNA after 48 h was observable. The reasons for the pathologic changes in lipogenic signaling observed in NAFLD liver samples are not fully understood [48]. A possible explanation may lie in the stress signaling. FFA overload induces oxidative and ER stress in hepatocytes. A recently published study by our group has shown that ER stress is even more pronounced under steatotic conditions [41], which in turn induces cleavage of SREBP-1c [49].

Overall, our data suggest that regulatory mechanisms to adapt metabolism to different levels of lipid accumulation work on different time scales. A study on mice fed a Western diet reported that mitochondrial adaptation in hepatocytes of steatotic animals required several weeks [50]. Since mitochondria are the key site of fatty acid degradation, long-term negative effects on PPARα-regulated processes are a logical consequence of persistent substrate overload. When using PHHs, the initial lipid load of the cells, which is dependent on the pre-existing hepatic steatosis of the donor, also has to be taken into account [41]. As shown by our group, hepatocytes initially containing high lipid levels displayed a long-ranging decrease in mitochondrial activity. Structural and functional impairments of mitochondria are proven consequences of hepatic steatotis [51].

### 3.3. Genistein Shows Hepatotoxic Effects at High Concentrations

Genistein is a naturally occurring isoflavone that has shown beneficial effects on lipid homeostasis. On the one hand, many of genistein’s positive properties are ascribed to its antioxidative capacities [27]. On the other hand, it can cause oxidative genetic damage itself. In colon carcinoma cells, 100 µM genistein induced DNA strand breaks [52]. In our assessment of genistein-induced hepatotoxicity, we observed a decreased cell viability at this concentration. Other groups, however, did not observe any impairment in cell viability after treatment with up to 100 µM in the human hepatoma cell line HepG2 [53,54], possibly due to a stronger resilience of the immortalized cell line. Another recent publication reported repeated viability assessments of genistein-treated HepG2 cells [55]. Here, no cytotoxic effect was seen after incubation with 100 µM for 24 h, but this became obvious after 48 h of incubation [55].

### 3.4. Genistein Acts as a PPARα Agonist Only in Nonsteatotic PHHs

Thus far, research on the beneficial effects of genistein in the context of NAFLD has mainly been conducted as in vivo experiments in rodents [30]. Some clinical data are available on genistein-treated patients featuring NAFLD or metabolic syndrome, indicating a balancing effect on lipid metabolism [56].

Genistein has been shown to be a PPARα agonist [53]. In our analyses, PHH incubation with the isoflavone led to a decrease in PPARα mRNA expression. A study on high-fat diet-fed mice also reported a tendency towards reduced PPARα mRNA levels after genistein treatment [57]. In contrast, our investigations showed a dose-dependent induction of PPARα protein expression. This effect was restricted to control PHHs, suggesting that FFA-treated PHHs differ in their susceptibility to PPARα stimulation. The steatotic PHHs already exhibited an upregulation of PPARα protein, which could not be further ameliorated by the addition of genistein. Asrih et al. made comparable observations after treatment of HepG2 cells with the metabolic regulator fibroblast growth factor 21 (FGF21) [58]. Individual incubation with either FFAs or FGF21 increased fatty acid oxidation and the mRNA level of the PPARα downstream target CPT1L. Upon simultaneous administration of FFAs and FGF21, HepG2 cells exhibited a similar response but no further increase, which was explained by a potential loss of FGF21 action in the presence of FFAs. The complex mechanisms regulating PPARα activation and turnover are still the subject of intense research [59]. There is increasing evidence on post-translational regulation of PPARα by miRNAs, which in turn are dysregulated in steatotic hepatocytes [60]. It was further shown that PPARα ligands are capable of preventing ubiquitination and subsequent degradation of the nuclear receptor via the proteasome [61]. Thus, one possible explanation for elevated protein levels despite reduced mRNA levels in the presence of genistein might be a prolongation of the PPARα protein half-life accompanied by an inhibition of its ligand-activated transcriptional capacity.

### 3.5. In Steatotic PHHs, Genistein Leads to the Downregulation of Activated SREBP-1c

Rodent in vitro and in vivo NAFLD models show a reversal of steatosis-induced SREBP1-c protein upregulation after treatment with genistein [29,62]. Additionally, in the human hepatoma cell line HepG2, genistein treatment reduces SREBP-1c protein, whereas its mRNA level is not affected. Since the ratio of mature to immature SREBP-1c was shown to decline, it was proposed that genistein reduces SREBP-1c proteolytic cleavage [63]. Our investigations confirm the ability of genistein at a dose of 10 µM to attenuate the upregulation of cleaved SREBP-1c in steatotic hepatocytes. In the control PHHs, genistein did not lead to a significant decrease in SREBP-1c protein. Transcription of FASN was not affected either. In the abovementioned NAFLD models with genistein administration, isoflavone treatment was conducted in parallel with the induction of steatosis. In our experimental setting, however, steatosis was first induced and then genistein was administered on steatotic cells. It might therefore be possible that further consequences of the treatment become visible after a prolonged observation period.

### 3.6. In Steatotic Hepatocytes, Genistein Suppresses De Novo Lipogenesis, While Genistein-Mediated Induction of Hepatic Lipid Degradation Is Abrogated

In summary, our data show that the in vitro model utilizing PHHs serves as a realistic approach for the study of early NAFLD development. FFA treatment activated β-oxidation, which could be observed by elevated PPARα protein levels and a subsequent increase in CPT1L and ACSL1 transcription. A similar induction of PPARα protein was also achieved by genistein treatment, but only in nonsteatotic PHHs. Conversely, the examined PPARα transcription targets were not influenced by genistein, and PPARα mRNA was even reduced. Considering the current literature on PPARα, we hypothesize that these diverging observations might be due to a stabilizing effect of the PPARα ligand genistein on the protein while simultaneously interfering with its ligand-activated transcription capacity. These responses were only present in nonsteatotic PHHs. Former FFA treatment abolished the genistein effect on PPARα. In contrast, abrogation of the de novo lipogenesis pathway via genistein-mediated suppression of SREBP-1c activation was only seen in steatotic hepatocytes. Long-term steatotic conditions in vivo lead to aberrations in the PPARα and SREBP-1c pathways [18,48,64]. Such changes were also confirmed in short-term in vitro investigations [29,64]. Our current and former observations, however, imply that the molecular manifestations of hepatic steatosis as seen in PHHs are not as fast-paced as those reported for hepatic cell line experiments. We have shown that the rate of lipid accumulation in PHHs depends on the initial lipid load and subsequently reaches a steady state [41]. Whether genistein can then interfere with and reverse the downregulation of PPARα would be an interesting matter for future studies on PHHs. Overall, we can confirm that genistein exerts beneficial effects on different levels of hepatic lipid metabolism and could thus be a promising drug candidate for NAFLD prevention and therapy.

### 3.7. Limitations

The strengths and limitations of our study are closely intertwined. Among the various monocellular in vitro models to study liver function and pathologies, PHHs are closest to the conditions found in reality. However, the cellular response in culture depends on the former health status of the donor, which is why PHHs do not behave as homogeneously as immortalized cell lines. In the present study, this circumstance is reflected by the fact that several results are associated with high standard deviations. Furthermore, the protocol for hepatocyte isolation requires a sufficiently large liver specimen; thus, sample material from surgical patients is scarce, and the number of biological replicates is limited.

Aside from that, several of our results suggest a time-dependent effect of the FFA treatment on the signaling pathways of PPARα and SREBP-1c. Yet, this study was not designed as a time course experiment and cannot provide sufficient insight into the question of time dependency. A study focusing on the time course of FFA-induced changes in PPARα and SREBP-1c signaling will therefore be a future goal of our group.

## 4. Materials and Methods

### 4.1. Isolation of Primary Human Hepatocytes

Liver tissues for hepatocyte isolation were obtained after informed consent was obtained from patients undergoing liver surgery at the Department of General, Visceral and Transplantation Surgery at Charité University Medicine Berlin, Germany. The study was conducted according to the Declaration of Helsinki and received prior approval from the local ethics committee (Charité University Medicine Berlin, registration number EA2/076/09, date 28 July 2019). Tissue samples were freshly retrieved from macroscopically tumor-free areas of the resected livers. Samples with infections with hepatitis B virus, hepatitis C virus or human immunodeficiency virus or higher grades of liver cirrhosis (Child-Pugh class B or C) were excluded. Isolation of PHHs was performed as previously described [65,66]. In brief, PHHs were isolated in a two-step EGTA/collagenase perfusion technique. After isolation, PHHs were pooled, washed with phosphate buffered saline (PBS; PAA Laboratories, Pasching, Austria) and subjected to density gradient centrifugation for 20 min at 1280 g at 4 °C using Percoll (Biochrom AG, Berlin, Germany). The resulting cell pellet was washed, and the cells were resuspended in PHH culture medium (Williams Medium E, 100 U/100 µM penicillin/streptomycin, 1 mM sodium pyruvate, 15 mM HEPES, 10% fetal bovine serum (FBS), 1% nonessential amino acids (MEM NEAA; all provided by Gibco Invitrogen, Karlsruhe, Germany), 1.6 µM dexamethasone (Fortecortin^®^, Merck, Darmstadt, Germany), and 1 mM human insulin (Sanofi-Aventis, Frankfurt a.M., Germany)) and seeded on cell culture plates at a density of 100,000 viable cells/cm^2^. The cell culture plates were previously coated with type I collagen that was prepared from rat tails in our own laboratory according to the protocol of Rajan et al. [67].

### 4.2. Cell Culture, In Vitro Induction of Steatosis and Genistein Treatment

PHH adherence was achieved overnight in PHH culture medium at 37 °C, and steatosis was induced according to the in vitro steatosis model described by Gómez-Lechón et al. [40]. PHH culture medium was replaced by control (Williams Medium E, 100 U/100 µM penicillin/streptomycin, 1 mM sodium pyruvate, 15 mM HEPES, 5% FBS, 1% MEM NEAA supplemented with 0.3% methanol), or FFA-containing medium (control medium supplemented with 1 mM oleate/palmitate in a 2:1 ratio (Gibco Invitrogen, Karlsruhe, Germany) in methanol (J.T. Baker, Deventer, The Netherlands)) and hepatocytes were cultured for 24 h. For genistein treatment, the respective cells were further incubated with control medium supplemented with 0, 1, 5, 10, 50, or 100 µM genistein (Roth, Karlsruhe, Germany) for another 24 h. Genistein was dissolved in dimethyl sulfoxide (DMSO, Sigma-Aldrich, Steinheim, Germany) and diluted with low FBS-PHH culture medium to the final concentrations (with a final DMSO concentration of 0.5%).

### 4.3. Evaluation of Steatosis by Oil Red O and Sulforhodamine B (SRB) Staining

Intracellular lipid accumulation was evaluated by staining with the diazo dye Oil Red O (Sigma Aldrich, Steinheim, Germany), which stains neutral lipids. Before staining, the hepatocytes were washed with PBS and fixed for 30 min with 3.7% formaldehyde (Herbeta Arzneimittel, Berlin, Germany). Cells were incubated with Oil Red O working solution (8.6 mM Oil Red O in isopropanol, diluted in dH_2_O in a 3:2 ratio) for at least 20 min. Unfixed dye was removed by thorough washing with tap water, and cells were dried at room temperature. Then, Oil Red O dye was dissolved from the cells with isopropanol (Roth, Karlsruhe, Germany), and the absorbance was measured at 492 nm with a spectral photometer (Fluostar Optima, BMG, Offenburg, Germany).

To calculate lipid content relative to cell count, SRB (Sigma Aldrich, Steinheim, Germany) protein staining followed the Oil Red O assay. SRB binds to protonated amino acids. The fixed hepatocytes were washed with PBS and incubated with SRB solution (0.4% (*m*/*v*)) for 30 min. Unbound SRB dye was removed by thorough washing with acetic acid (1% (*v*/*v*), Merck, Darmstadt, Germany). A 10 min incubation with 10 mM Tris solution dissolved the bound SRB. Absorbance was measured with a spectral photometer at 580 nm.

### 4.4. XTT Assay

Cell viability was assessed using the Cell Proliferation Kit II (XTT; Roche Diagnostics, Mannheim, Germany) according to the manufacturer’s instructions. The yellowish tetrazolium salt XTT (2,3-bis-(2-methoxy-4-nitro-5-sulfophenyl)-2*H*-tetrazolium-5-carboxanilid) is converted in the mitochondria of viable cells to orange formazan. In brief, XTT labeling reagent was mixed with XTT electron-coupling reagent in a 50:1 ratio. Fifty microliters of the mixture were added to each well of a 96-well plate, followed by incubation for 24 h. Absorbance was measured at 492 nm using a spectral photometer.

### 4.5. LDH, AST and Urea Assays

Lactate dehydrogenase (LDH) and aspartate aminotransferase (AST) are intracellular enzymes that are released upon cell membrane damage. To evaluate membrane integrity, the enzymatic activity of LDH and AST was measured in the cell culture supernatant utilizing Fluitest^®^ reaction kits (Analyticon, Lichtenfels, Germany).

Urea is the end product of amino acid catabolism and results from the conversion of toxic ammonia in the urea cycle, which occurs mainly in the liver. Therefore, the metabolic activity of hepatocytes can be assessed by the amount of urea released into the cell culture supernatant. The urea concentration was measured with a Fluitest^®^ reaction kit.

All three assays were performed according to the manufacturer’s protocol. Absorbance was measured at 340 nm with a spectral photometer.

### 4.6. RT-qPCR Analyses

Messenger RNA expression levels of PPARα, SREBP-1c, ACSL1, CPT1L, and FASN were analyzed by RT-qPCR. Total RNA was isolated from hepatocytes by Trizol^®^ (Invitrogen, Karlsruhe, Germany) extraction according to the manufacturer’s protocol. RNA concentrations were determined with a spectral photometer (Nanodrop ND-1000, Peqlab, Erlangen, Germany) after resuspension with 50 µL diethylpyrocarbonate (DEPC) H_2_O (Roth, Karlsruhe, Germany). Only samples with an A260/A280 ratio of at least 1.9 were processed further. If subsequent experiments were not performed immediately, the samples were stored at −80 °C.

Reverse transcription was conducted with the cDNA Synthesis Kit by Fermentas (St. Leon-Rot, Germany) adhering to the manufacturer’s protocol. The kit utilizes Oligo(dT)_18_ primers. For each reaction, 1 µg total RNA was used. The cycling profile used in the Veriti™ 96-well thermocycler (Applied Biosystems, Foster City, CA, USA) is detailed in Table 1. The samples were immediately processed further or stored at −20 °C for a maximum of 14 d.

qPCR was performed with a Step One Plus Real Time PCR Cycler (Applied Biosystems, Foster City, CA, USA) with 50 ng cDNA used for each reaction. Specific hydrolysis probes for the PPARα, SREBP-1c, ACSL1, CPT1L, and FASN genes and TaqMan^®^ Gene Expression Master Mix were purchased from Applied Biosystems (Foster City, CA, USA). The β-actin gene (ACTB) served as the reference gene. Assay details are listed in Table 2. All samples were measured in duplicates. The qPCR reaction profile is detailed in Table 3. Relative gene expression was calculated using the comparative C_T_ method (2^−ΔΔCT^ method).

### 4.7. Western Blot Analyses

Proteins were extracted utilizing the Subcellular Protein Fractionation Kit by Thermo Scientific (Rockford, IL, USA). To each cell culture dish containing approximately 2.1 × 10^6^ viable PHHs, 200 µL of cytosolic extraction buffer was added. The subsequent steps followed the manufacturer’s instructions, resulting in distinct fractions of cytoplasmic, membrane-bound and nuclear soluble protein extractions for each sample. For protein quantification, the bicinchoninic acid assay (BCA; Interchim, Montelucon, France) was used according to the manufacturer’s protocol. Samples were adjusted to a protein quantity of 1.2 µg/µL with dH_2_O and 20% sample buffer (0.4 M Tris base, 10% SDS, 50% glycerol, 0.025% bromphenol blue and 25% mercaptophenol, all from Sigma-Aldrich, Steinheim, Germany) and incubated at 100 °C for 5 min. For each sample, 30 µg protein was separated at a continuous voltage of 120 V on an 8% SDS-polyacrylamide gel. Then, protein transfer to a nitrocellulose membrane (Bio-Rad Laboratories Inc., Munich, Germany) was carried out with a tank blotting system (Mini Trans-Blot^®^ Cell and Module; Bio-Rad Laboratories Inc., Munich, Germany) applying an electric current of 340 mA for 60 min. After staining of the protein bands with Ponceau S solution (5 g Ponceau S in 75 g trichloroacetic acid (both by Merck, Darmstadt, Germany) and 75 g sulfosalicyclic acid (Sigma-Aldrich, Steinheim, Germany) in 250 mL dH_2_O) and two washing steps with dH_2_O, the membrane was blocked with 5% nonfat dry milk (Applichem, Darmstadt, Germany) in TBST (0.1% Tween 20 (Merck, Darmstadt, Germany) in 20% TBS (50 mM Tris base (Sigma-Aldrich, Steinheim, Germany) and 150 mM sodium chloride (Merck, Darmstadt, Germany) in dH_2_O at pH 7.5) by continuously shaking for 1 h at room temperature. Afterwards, the membrane was washed with TBST and incubated with specific primary antibodies against PPARα, SREBP-1c, α-tubulin, p84, or Na^+^/K^+^-ATPase at 4 °C. p84 served as a reference protein for nucleic PPARα or SREBP-1c, α-tubulin as a reference protein for cytosolic PPARα and Na^+^/K^+^-ATPase for ER membrane-bound SREBP-1c. Four washing steps with TBST were followed by incubation with the secondary antibodies at RT. Antibodies were diluted in TBST and 1% (*v*/*v*) bovine serum albumin (BSA; Sigma-Aldrich, Steinheim, Germany). Further details on the primary and secondary antibodies are listed in Table 4. Prior to detection, the membrane was again washed four times with TBST and then incubated with ECL™ Detection Reagents (Sigma-Aldrich, Steinheim, Germany) according to the manufacturer’s instructions. Chemiluminescence was detected with a VersaDoc Model 4000 (Bio-Rad Laboratories Inc., Munich, Germany).

### 4.8. Statistical Analyses

Values are expressed as the mean + SD. Each value represents the mean of at least three biological replicates. GraphPad Prism 7 (San Diego, CA, USA) was employed for significance analyses and chart design. Significant differences between single values were analyzed by a paired t-test. For significance testing among multiple groups, a repeated measures two-way ANOVA (with the two factors of genistein treatment and steatotic treatment) was employed followed by post hoc tests to correct for multiple comparisons (Tukey test for the comparison of genistein treatment among steatotic and nonsteatotic PHHs or Sidak correction for differences between the steatotic and nonsteatotic groups treated with the same genistein dose). Statistical significance was assumed at *p* ≤ 0.05.

## Figures and Tables

**Table 1:** Thermocycling profile of the reverse transcription reaction.

Step	Temperature ( °C)	Time (min)
Annealing	65	5
Reverse transcription	37	60
Inactivation	70	5

**Table 2:** TaqMan^®^ gene expression assays (Applied Biosystems, Foster City, CA, USA) applied for qPCR.

Gene Symbol	Gene Name	Assay ID
PPARA	Peroxisome proliferator activated receptor alpha	Hs00947539_m1
SREBP-1c	Sterol regulatory element binding transcription factor 1 (SREBF1)	Hs01088691_m1
CPT1L	Carnitine palmitoyltransferase 1A (CPT1A)	Hs00912671_m1
ACSL1	Acyl-CoA synthetase long-chain family member 1	Hs00242530_m1
FASN	Fatty acid synthase	Hs01005622_m1
ACTB	Actin beta	Hs99999903_m1

**Table 3:** qPCR thermocycling conditions.

Step	Temperature ( °C)	Time (min:s)	Cycles
UNG ^1^ incubation	50	2:00	1
Polymerase activation	95	10:00	1
Denaturation	95	0:15	40
Annealing/extension	60	1:00	

^1^ Uracil-*N*-glycosylase.

**Table 4:** Antibodies for Western blotting.

Antibody (Manufacturer)	Dilution	Incubation Time
Mouse PPARα (Dianova, Hamburg, Germany)	1:1000	Overnight
Mouse SREBP-1c (Biozol, Eching, Germany)	1:200	2 h
Mouse α-tubulin (Sigma-Aldrich, Steinheim, Germany)	1:2000	1 h
Mouse p84 (Abcam, Cambridge, UK)	1:2000	1 h
Rabbit Na^+^/K^+^-ATPase (Cell Signaling, Danvers, MA, USA)	1:1000	1 h
Sheep anti-mouse (Amersham, Freiburg, Germany)	1:4000	1 h
Donkey anti-rabbit	1:4000	1 h

## Data Availability

The data presented in this study are available on request from the corresponding author. The data are not publicly available due to data protection regulations.

## Supplementary Materials

The following are available online, Figure S1: Evaluation of the effect of genistein on the transcriptional targets of PPARα and SREBP-1c in steatotic PHHs, Table S1: Details on statistics for Figure 5, Figure 6 and Figure S1.

## Abbreviations

ACC	acetyl-CoA carboxylase
ACSL1	long-chain acyl-CoA synthetase 1
acyl-CoA	acyl-coenzyme A
ACTB	β-actin gene
ANOVA	analysis of variance
AST	aspartate transaminase
BCA	bicinchoninic acid assay
cDNA	complementary deoxyribonucleic acid
CPT1A	carnitine palmitoyltransferase 1A
CPT1L	carnitine palmitoyltransferase 1L
dH_2_O	distilled water
DEPC	diethylpyrocarbonate
DMSO	dimethyl sulfoxide
EGTA	ethylene glycol tetraacetic acid
ER	endoplasmatic reticulum
FASN	fatty acid synthase
FBS	fetal bovine serum
FFA	free fatty acid
FGF21	fibroblast growth factor 21
LDH	lactate dehydrogenase
NAFLD	nonalcoholic fatty liver disease
miRNA	micro ribonucleic acid
mRNA	messenger ribonucleic acid
Na^+^/K^+^-ATPase	sodium–potassium adenosine triphosphatase
NASH	nonalcoholic steatohepatitis
PBS	phosphate buffered saline
PHH	primary human hepatocyte
PPARa	peroxisome proliferator activated receptor alpha
qPCR	quantitative polymerase chain reaction
RT	room temperature
RT-qPCR	reverse transcription-quantitative polymerase chain reaction
SD	standard deviation
SDS	sodium dodecyl sulfate
SRB	sulforhodamine B
SREBF1	sterol regulatory element binding transcription factor 1
SREBP-1c	sterol regulatory element-binding protein 1c
TBS	tris-buffered saline
Tris	tris(hydroxymethyl)aminomethane
UNG	uracil-N-glycosylase
XTT	2,3-bis-(2-methoxy-4-nitro-5-sulfophenyl)-2H-tetrazolium-5-carboxanilid
