# Supplementary material for: Influence of Genistein on Hepatic Lipid Metabolism in an In Vitro Model of Hepatic Steatosis
Source: Molecules. 2021 Feb 22;26(4):1156. doi: 10.3390/molecules26041156 (PMC7926972; doi:10.3390/molecules26041156)
Supplement: Supplementary file 1 [file molecules-26-01156-s001.zip › molecules-1085947-supplementary.pdf]

molecules

Supplementary Materials

Influence of Genistein on the Hepatic Lipid Metabolism in an In Vitro Model of Hepatic Steatosis

Lena Seidemann [1,2], Anne Krüger [3], Victoria Kegel-Hübner [1], Daniel Seehofer [1,3] and Georg Damm [1,2,3,*]

[1] Department of Hepatobiliary Surgery and Visceral Transplantation, University Hospital, Leipzig University, 04103 Leipzig, Germany; lena.seidemann@medizin.uni-leipzig.de (L.S.); victoria.kegel@yahoo.de (V.K.-H.); daniel.seehofer@medizin.uni-leipzig.de (D.S.)
[2] Saxonian Incubator for Clinical Translation (SIKT), Leipzig University, 04103 Leipzig, Germany
[3] Department of General, Visceral- and Transplantation Surgery, Charité University Medicine Berlin,13353 Berlin, Germany; kruegera@uni-greifswald.de. (A.K.)
* Correspondence: georg.damm@medizin.uni-leipzig.de; Tel.: +49-341-9739656 (G.D.)

Supplementary Figures

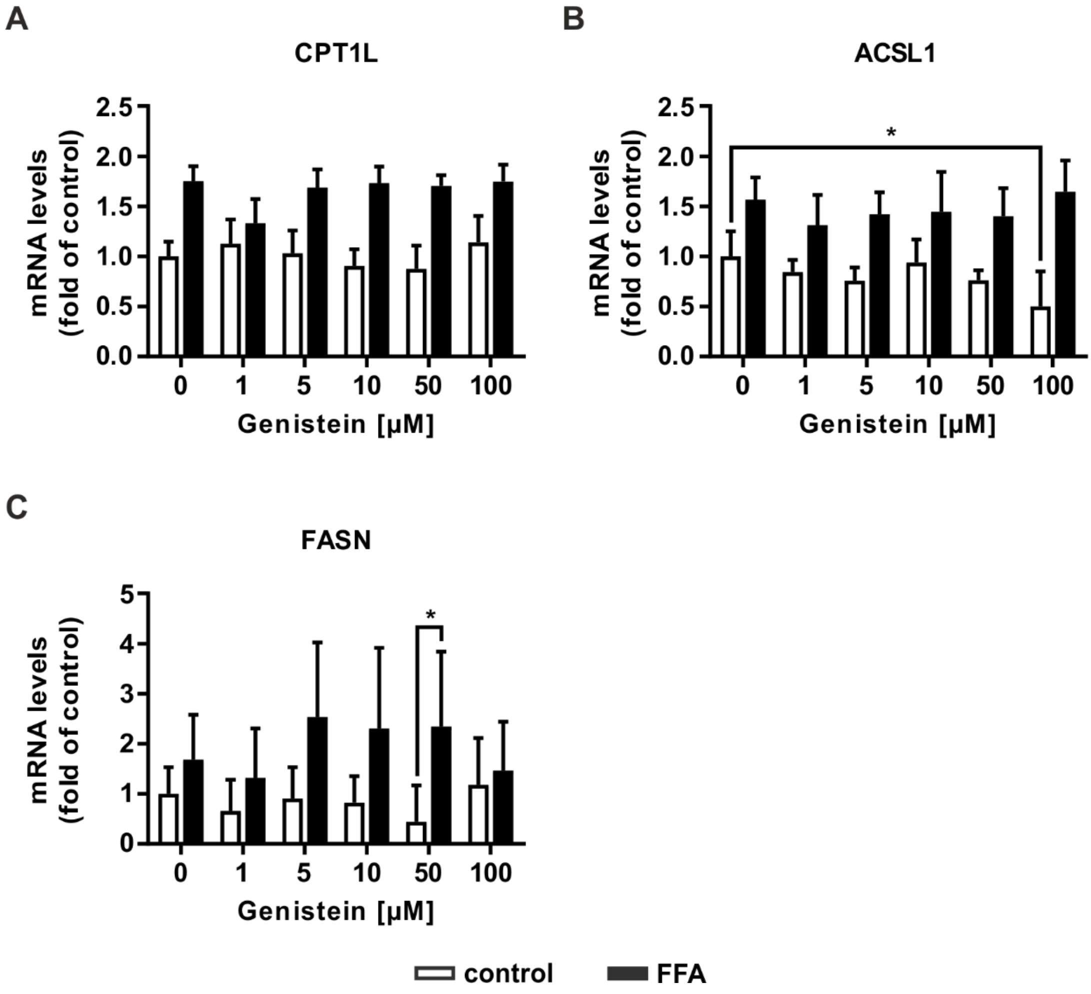

Figure S1. Evaluation of the effect of genistein on the transcriptional targets of PPARα and SREBP-1c in steatotic PHHs. PHHs were treated with 1 mM FFA for 24 h, followed by 24 h of additive treatment with genistein (0, 1, 5, 10, 50, 100 µM). Relative expression levels of (A) CPT1L, (B) ACSL1 and (C) FASN mRNA were determined by RT-qPCR. Data are shown as mean + SD, n = 5, two-way ANOVA with Tukey or Sidak post hoc test. $p \leq 0.05$ (*). Selected comparisons are shown; for details on the statistical evaluation, see Table S1C.
